# Errata

**Published:** 1784

**Authors:** 


					) ...
ERRATA.
In t!*e 4th volume, page 431, for intcgr itatx read integritate?

				

